# Brice Pitt, MD, FRCPsych

**DOI:** 10.1192/bjb.2021.56

**Published:** 2021-12

**Authors:** Claire Hilton

Formerly Emeritus Professor of Psychiatry of Old Age, Imperial College, London, UK

**Figure:**
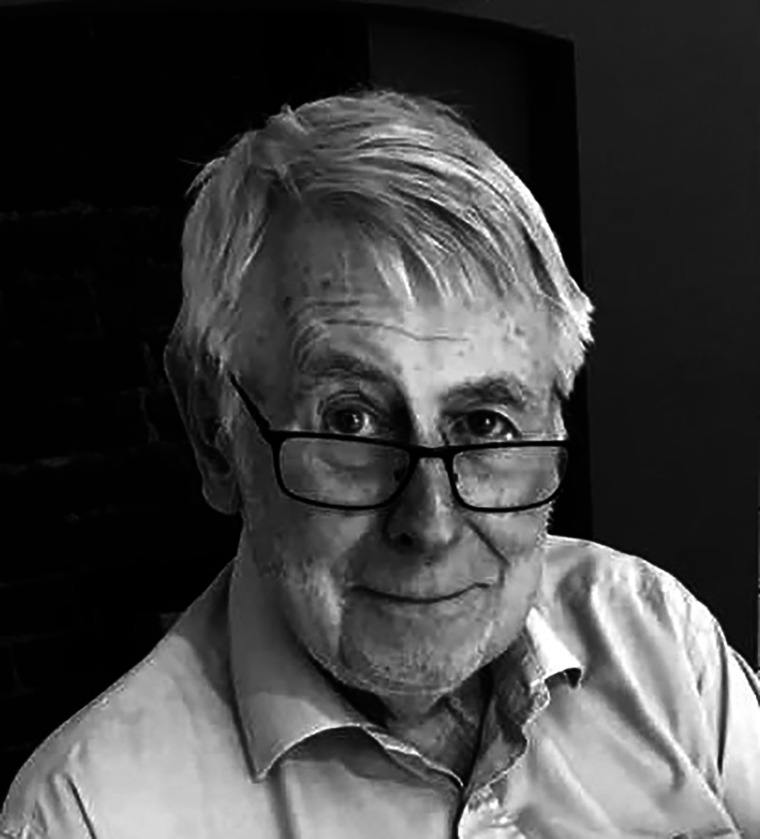

Brice Pitt, who died aged 89 on 16 January 2021, was a highly significant pioneer of old age psychiatry, as well as a colourful personality. In 1971, he was a founder member of the informal ‘coffee house’ group of psychogeriatricians, which preceded the RCPsych's Special Interest Group (now a Faculty) for Old Age Psychiatry. He was the group's first secretary (1973–1978). As the chair of the subsequent Section (1986–1990), he led old age psychiatry through turbulent debate to achieve government recognition as a distinct medical specialty in 1989. He also chaired the College's Public Education Committee and, associated with this role, wrote *Down with Gloom! or How to Defeat Depression* (illustrated by Mel Calman). In it, as elsewhere in his writing, he was open about his personal experience of depression.

Brice's passion for his subject was evident in *Psychogeriatrics: An Introduction to Psychiatry of Old Age* (1974). About this book, he said: ‘I wrote down everything I knew off the top of my head. It was a doddle’. His turn of phrase was often vivid and insightful: psychogeriatricians were ‘a happy band of pilgrims’ who needed ‘occasional militancy […] to gain a fair share of scant resources, to put them to best use, to make do with too little while wheeling, dealing, and fighting for more’. *Psychogeriatrics* was followed by other books, including *Feelings about Childbirth* (1978), which was based on his MD thesis on post-partum depression (a life-long interest: he was President of the Association for Postnatal Illness at the time of his death) and *Mid-Life Crisis: Its Cause and How to Overcome It* (1980), and they all received international acclaim. These volumes were variously translated into Japanese, Spanish, Polish, Finnish, French, Croatian, Afrikaans and other languages.

Brice was born on 19 December 1931, the son of Norman Pitt, a surgeon, and Emily (née Crawford), a nurse. Brought up in Surrey, he attended Epsom College. In the immediate post-war years, children perhaps conformed more to parental direction than they do today. Brice wanted to be an actor, but his father told him he would have to study medicine. He was unsure how to reconcile this direction with his wishes, but when watching Alfred Hitchcock's psychiatric melodrama *Spellbound* (1945), he decided that if medicine was to be his destiny, he would be a psychiatrist.

Like his father, Brice went to Guy's Hospital Medical School in London. David Stafford-Clark, psychiatrist, television personality and author of books on psychiatry for a lay readership, was his teacher. After house jobs came National Service in the Army, first at The Royal Victoria Military Hospital at Netley, then Singapore and Hong Kong. In 1960, Major Brice Pitt moved back into civilian medicine. He opted for psychiatry training at Springfield Hospital in south-west London. There, he treated his ‘first proper old person’. He was ‘perplexed by the richness of the symptomatology’ and perturbed by the attitude that the hospital was ‘like a castle, a good registrar would fend off the elderly, as those who got in were bound to stay […] dumped by their family’. He objected to excluding people on account of age. Perhaps a childhood memory haunted him: ‘My sainted grandmother had a stroke and threw a knife at me in a tantrum: this affected me profoundly and left me wondering why’.

As a senior registrar, Brice worked with Ford Robinson, who had a ward and day hospital for functionally ill older people at St Clements Hospital, Mile End, London. It was rare in those days to have any dedicated psychogeriatric training. Brice also trained at Claybury, a large psychiatric hospital also in London. It had a ‘tremendous atmosphere emanating from the therapeutic community approach’ nurtured by the ‘superb’ physician superintendent Denis Martin. Brice called Claybury ‘Camelot’, after the mythical fellowship of King Arthur, with all participants equal at the Round Table.

Appointed consultant psychogeriatrician at Claybury in 1966, Brice inherited 400 occupied beds, a catchment area of about 65 000 over-65s, a social worker, a senior house officer and some general practitioner sessions. Local geriatrician Malcolm Hodkinson said to him: ‘You don't want a waiting list, you never admit for continuing care, and you don't want to run a sleepy service’, and he thought: ‘My God, the one thing I do not want ever to be accused of by Malcolm is running a sleepy service’. And he never did. He started by establishing a mixed-sex ward at Claybury, risqué in a gender-segregated hospital. He also introduced routine home assessments for older people, a practice that became widespread. Camelot fell when Martin died. New-style district general hospital psychiatry beckoned, and a prize post at the brand-new Princess Alexandra Hospital, Harlow, lured Brice back to general psychiatry. But in 1971 he returned to psychogeriatrics in Tower Hamlets, where innovation included a collaborative psychiatric–geriatric unit with geriatrician Chris Silver.

In the early 1980s, Brice moved to Barts (City and Hackney), to join psychogeriatrician Alan Gardner, ‘a very obsessional serious fellow, while there is something a bit flighty about me some of the time’, he said. In 1986, he moved again, to St Mary's Hospital, Paddington, as the UK's fourth professor of old age psychiatry. While there, he established the Hammersmith Hospital memory clinic, one of the first in the country. This led to him being dubbed ‘Memory Man’ in a local newspaper, for his pioneering work.

Brice also fulfilled his dream to be an actor. With the Tower Theatre Company, he played royalty, noblemen, judges, bishops and Badger in *The Wind in the Willows*. He directed plays and wrote some, including *Anatomy of Melancholy* and *The Memory Clinic*. In 2005, under the pseudonym Beric Norman, he wrote a novel, *Mordred's Version*, rooted in the original Camelot.

Chaucer would have appreciated his words and humour at the RCPsych's Old Age Faculty meeting in 2013, when he was presented with its lifetime achievement award:

In March, when Spring is nigh, but strong winds blow
The Faculty of Old Age Shrinken blithely go
On Pilgrimage, to venues academic
And each and every one a well trained medic
To discourse on derangements of the mind
Of older persons, troubled with all kind
States of confusion, madness, melancholy –
Yet to my mind the meeting was full jolly […]
His presence at the gathering, to receive meant
His peers’ award for his Lifetime Achievement! […]
Brice was delighted to be offered this award
Which he never knew existed: Thank you, Lord!^1^

Brice had a large family. Married three times, he was father of Gareth, Caroline, Tristram and Rosalind, grandfather of Shem, Fiona, Helen and Joshua, and stepfather to children from the marriage of his third wife, Judy. They all survive him.
